# Insulin Sensitivity and Beta-Cell Function Are Associated with Arterial Stiffness in Individuals without Hypertension

**DOI:** 10.1155/2013/151675

**Published:** 2013-02-26

**Authors:** Chuchen Meng, Min Sun, Zhixiao Wang, Qi Fu, Mengdie Cao, Zhenxin Zhu, Jia Mao, Yun Shi, Wei Tang, Xiaoping Huang, Yu Duan, Tao Yang

**Affiliations:** Department of Endocrinology, The First Affiliated Hospital of Nanjing Medical University, 300 Guangzhou Road, Nanjing, Jiangsu 210029, China

## Abstract

*Aim*. We investigated the relationship between brachial-ankle pulse wave velocity (baPWV) and glucose levels, insulin sensitivity, and beta-cell function in Chinese individuals with or without hypertension. *Methods*. We recruited 3137 nondiabetic individuals whose age, body mass index (BMI), glucose levels, blood pressure (BP), lipids, hemoglobin A1C (HbA1c), baPWV, and insulin levels were measured. *Results*. In normotensive group, 2 h glucose levels (*β* = 0.046, *P* < 0.001) associated with baPWV, showed a significant increase in patients with NG as compared to those with DM (*P* = 0.032). The hypertensive group showed no such differences. The Matsuda index (*β* = 0.114, *P* < 0.001) and HOMA-**β** (*β* = 0.045, *P* < 0.001) were negatively correlated with baPWV while lnHOMA-IR (*β* = 0.196, *P* = 0.076) and the Quantitative Insulin Sensitivity Check Index (QUICKI) (*β* = 0.226, *P* = 0.046) showed a borderline negative correlation. BaPWV significantly decreased (*P* = 0.032) with an increase in insulin sensitivity in individuals with both normal BP and glucose tolerance. *Conclusions*. BaPWV was significantly associated with 2 h glucose levels, insulin sensitivity and beta-cell function in normotensive population, whereas in hypertensive individuals, BP was the dominant factor influencing arterial stiffness. Individuals with abnormal insulin sensitivity in the absence of diabetes and hypertension are also at an increased risk of arterial stiffness.

## 1. Introduction

It is well known that the prevalence of cardiovascular disease (CVD) is high among individuals with abnormal glucose tolerance, resulting in significant mortality and morbidity rates from macrovascular diseases in such individuals. A large number of population studies have focused on the relationship between arterial stiffness and glucose abnormality; however, the conclusions remain controversial. Among these studies, most have revealed a positive association between arterial stiffness and type 2 diabetes [1–4], while some have found fasting and postchallenge glucose levels and HbA1c levels, which reflect 2-3 months of glycemic control, to be independently related to accelerated stiffening of arteries [4–6]. However, the best predictor of arterial stiffness among these remains unclear. Some studies show that 2 h blood glucose levels are better predictors than fasting blood glucose levels and HbA1c levels [7, 8], while another study reported different results [6]. In addition, recent studies have suggested that 1 h or 30 min blood glucose levels during the oral glucose tolerance test (OGTT) may also demonstrate a stronger correlation with arterial stiffness [9–11]. Variability in populations and evaluated factors between different studies may have led to these inconsistencies.

Indeed, individuals with CVDs and hypertension, who also commonly display abnormal glucose metabolism [12], individuals were rarely included in these studies. Therefore, we wanted to determine whether plasma glucose levels had the same effects on arterial stiffness in individuals with and without hypertension as well as elucidate the glycemic status that exerted maximum influence on arterial stiffness. Measuring pulse wave velocity (PWV) is widely used to assess arterial stiffness in the clinical setting because of its convenience and noninvasiveness [13]. Recent studies showed that PWV, especially brachial-ankle PWV (baPWV), can be used as a tool for screening cardiovascular risk and as a marker for assessing the severity of atherosclerotic vascular damage in the general population [14]. baPWV has been shown to be related with several factors such as age [15], blood pressure (BP) [16], dyslipidemia [17], and obesity [18]. Increased BP and glucose tolerance are also associated with increased insulin resistance (IR); however, few reports have described the relationship between IR and PWV. Therefore, we investigated the relationship between baPWV and glucose metabolism in a large sample of Chinese individuals with or without hypertension and delineated the impact of different glucose metabolism conditions on baPWV as well as the impact of the interaction between glucose levels and BP on arterial stiffness.

## 2. Subjects and Methods

### 2.1. Study Population

We recruited community-dwelling individuals aged 40–75 years living in the Gulou district, Nanjing, Jiangsu Province, China, from June 2011 to December 2011. A total of 10027 participants were invited by telephone or door-to-door visit to take part in this study. The protocol was approved by the ethics committee of The First Affiliated Hospital with Nanjing Medical University. After excluding individuals with a history of type 2 diabetes and cerebral, cardiovascular, peripheral artery, liver, and chronic kidney diseases, 3137 individuals were included in our study. The use of antihypertensive agents was also an exclusion criteria for our study.

### 2.2. Clinical and Biochemical Measurements

All measurements and procedures were performed from 7:30 to 11:30 AM following an overnight fast. The heights and weights of the subjects were measured with individuals dressed in only a single layer of clothes without shoes. Body mass index (BMI) was expressed as the weight in kilograms divided by the height in meters (kg/m^2^). The waist circumference was measured in the standing position at the level of the umbilicus by traditional examiners, and the waist-to-hip ratio (WHR) was calculated by dividing waist circumference by hip circumference. Fasting parameters, including levels of fasting plasma glucose (FPG), HbA1c, triglyceride (TG), low-density lipoprotein (LDL), high-density lipoprotein (HDL), and total cholesterol, were measured in each subject using standardized procedures. A 75 g OGTT was performed with plasma glucose sampling done at 0, 30, and 120 min. HbA1c levels in peripheral blood were measured using high-performance liquid chromatography (HPLC, BIO-RAD Company, USA). Plasma glucose levels were measured using the hexokinase method, HDL levels using the accelerator selective detergent method, LDL levels using the liquid selective detergent method, triglyceride (TG) levels using the glycerol phosphate oxidase method, and total cholesterol levels using the enzymatic method. All measurements were analyzed by the autoanalyzer (Modular E170; Roche). Serum insulin concentrations were determined with the Phadebas Insulin Test (Pharmacia, Uppsala, Sweden) using a radioimmunosorbent technique. Glucose metabolic status was categorized as follows: normal glucose tolerance (NGT; fasting glucose, <5.6 mmol/L; 2 h glucose, <7.8 mmol/L), prediabetes mellitus (pre-DM; including impaired fasting glucose: 5.6 mmol/L ≤ fasting glucose < 7 mmol/L or impaired glucose tolerance: 7.8 mmol/L ≤2 h glucose < 11.1 mmol/L, among them 143 participants had both IFG and IFG in this group), and diabetes mellitus (DM; fasting glucose ≥ 7.0 mmol/L or 2 h glucose ≥ 11.1 mmol/L).

Blood pressures (BP) was measured using a standardized sphygmomanometer and quantified from the mean of 3 consecutive measurements taken with at least 5 min intervals of rest. Hypertension was diagnosed if the average systolic BP (SBP) was ⩾140 mmHg or diastolic BP (DBP) was ⩾90 mmHg. BaPWV was measured in the supine position using a waveform analyzer (VP-1000; Colin Co., Komaki, Japan) after at least 5 min of bed rest. Briefly, the PWV value was calculated as the distance/transit time ratio and expressed in centimeter per second (cm/s). The mean of the left and right baPWV (average baPWV) was obtained for all participants and used for analysis.

### 2.3. Statistical Analysis

IR was calculated using the mathematical formula of HOMA-IR by dividing the product of fasting blood insulin (FBI; lU/mL) and fasting blood glucose (FBG; mmol/L) by 22.5. The Quantitative Insulin Sensitivity Check Index (QUICKI) was also used for the measurement of IR by dividing 1 by the sum of the logarithms of FBI and FBG [18]. The Matsuda Insulin Sensitivity Index (Matsuda ISI), which measures hepatic and peripheral IR, was calculated by the following formula: 10000/(FBG × FBI × mean glucose during OGTT × mean insulin during OGTT) [19]. Beta-cell function was estimated by the following formula: (20 × insulin (mU/L))/(glucose (mmol/L) − 3.5)). Collated data were analyzed using SPSS 17.0 for Windows (Chicago, IL, USA). Data from individual subjects was expressed as mean ± standard deviation, and the differences between groups were compared by Student's *t-*test. *χ* ^2^-statistic was used to compare proportions. Stepwise multiple linear regression analysis was performed to determine the effect of study variables on the PWV value. HOMA-IR was not normally distributed, so we transformed the variables logarithmically for all multivariate analyses. Comparisons of means were analyzed by one-way analysis of variance (ANOVA). A *P* value of <0.05 was considered statistically significant.

## 3. Results

### 3.1. Baseline Characteristics of Subjects

Table 1 shows the biochemical and clinical variables of the groups classified by glucose tolerance. All parameters differed significantly among the three groups. Among them, age and HDL, LDL and total cholesterol levels did not differ between the pre-DM and DM groups. The pre-DM group had a higher baPWV compared with the normal group, while the DM group had the highest baPWV.

### 3.2. Relationship between baPWV and Other Variables

Multiple regression analyses were performed to determine the relationship between each variable and baPWV value for the overall population and for the groups subdivided according to BP.  Table 2 shows that age (*β* = 0.355, *P* < 0.001), working heart rate (WHR) (*β* = 0.078, *P* < 0.001), heart rate (HR) (*β* = 0.171, *P* < 0.001), SBP (*β* = 0.489, *P* < 0.001), TG (*β* = 0.045, *P* = 0.001) levels, and 2 h plasma glucose levels (*β* = 0.052, *P* = 0.008) were positively associated with baPWV, while BMI (*β* = −0.086, *P* < 0.001) was negatively associated with baPWV. After dividing the subjects into two groups, the result displays that besides the aforementioned variables, HDL (*β* = −0.038, *P* = 0.037) levels were also associated with baPWV when BP was normal. In contrast, only age (*β* = 0.523, *P* < 0.001), BMI (*β* = −0.149, *P* = 0.007), HR (*β* = 0.243, *P* < 0.001), SBP (*β* = 0.474, *P* < 0.001), and TG (*β* = 0.097, *P* = 0.004) levels were still significantly related to baPWV in the individuals with hypertension.

### 3.3. Association between baPWV and Glucose Status in Individuals with or without Hypertension

**Figure 1:**
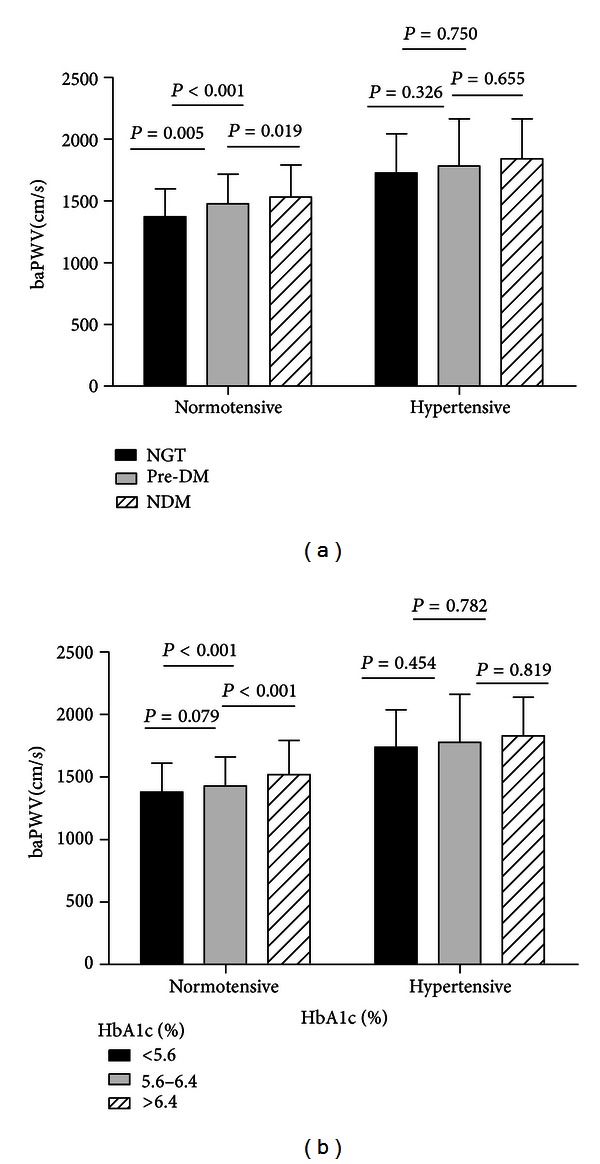
(a) Difference in PWV values across the glucose tolerance groups, after adjustment for age, gender, BMI, WHR, TG, lipids, HR, and MAP. *P* for trend = 0.000, 0.429 in normotensive and hypertensive groups, respectively. (b) Difference in PWV values across the HbA1c groups, after adjustment for age, gender, BMI, WHR, TG, lipids, HR, and MAP. *P* for trend = 0.001, 0.761 in normotensive and hypertensive groups, respectively.

We divided the subjects with different BPs into subgroups on the basis of diagnostic criteria or HbA1c levels, which represent glucose control in the last 3 months. With regard to the normotensive individuals, after adjustment for age, gender, BMI, lipids levels, HR, SBP, DBP and WHR, Figure 1(a) shows that the pre-DM group had a higher baPWV compared with the NGT group (*P* = 0.005), while the DM group had the highest baPWV among the three groups (NGT versus DM, *P* < 0.001; pre-DM versus DM, *P* = 0.019). When the diagnostic criteria were replaced by HbA1c levels, similar differences were found among the three groups. However, the difference between the NGT and pre-DM groups was not significant (NGT versus pre-DM, *P* = 0.079). On the other hand, no differences were observed for the hypertensive individuals, irrespective of the glucose metabolism status.

### 3.4. Independent Association of baPWV with the Indices of IR, Insulin Sensitivity, and Beta-Cell Function

As shown in Table 3, the Matsuda index (*β* = −0.114, *P* < 0.001) and HOMA-*β* (*r* = −0.045, *P* < 0.001) were negatively correlated with baPWV after adjustment for age, gender, BMI, WHR, lipids, HR, and MAP, while lnHOMA-IR (*β* = 0.196, *P* = 0.076) and QUICKI (*β* = 0.226, *P* = 0.046) showed a borderline negative correlation with baPWV. Only Matsudas index was weakly but significantly associated with baPWV in the hypertensive group (*β* = −0.126, *P* = 0.046).

**Figure 2:**
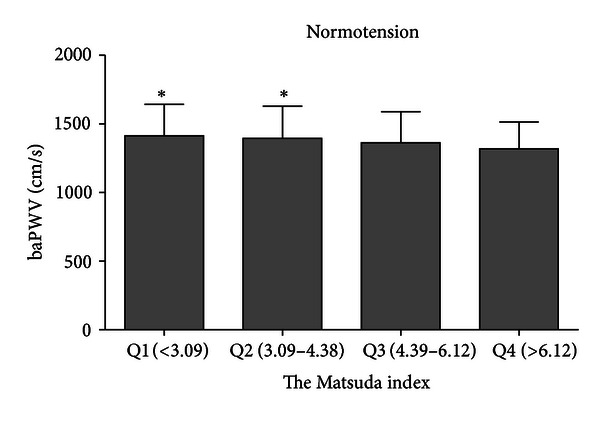
Difference in PWV values across the quartile of Matsuda's index among nondiabetic people in normotensive group, after adjustment for age, BMI, WHR, lipids, HR, and MAP. *P* for trend = 0.032; *P < 0.05 versus Q4.

We further selected individuals with NGT from the normotensive population and grouped them according to the quartile of the Matsuda index. BaPWV significantly decreased with an increase in insulin sensitivity (*P* = 0.032) (Figure 2).

## 4. Discussion

In the present study that involved individuals with no history of diabetes or hypertensive medication, 2 h glucose was positively and independently associated with baPWV, even after adjusting for confounding factors such as age, BP, and lipid profile. These data are in agreement with a number of studies. In a study based on five cohorts of Asian origin, postchallenge glucose was found to be an independent predictor of CVD, and 2 h plasma glucose levels were a superior predictor of immature death compared with FPG levels [18]. Similarly, a study involving a nondiabetic population demonstrated that 2 h plasma glucose levels were more strongly associated with cardiometabolic risk factors and subclinical atherosclerosis compared with FPG or HbA1c levels [7].

Hypertension is one of the major risk factors for arterial stiffness, and previous studies [20–22] have shown that PWV was higher in hypertensive individuals than in individuals with normal BP. However, previous studies have rarely focused on the interaction of hypertension and glucose metabolism with baPWV. To investigate whether an association exists between 2 h glucose levels and baPWV among individuals with different BP conditions, we divided the subjects into two groups on the basis of the diagnosis of hypertension. Separate analysis of the population via multiple linear stepwise analysis, including compounding variables, revealed that 2 h glucose levels were related to baPWV in the normal group but not in the hypertensive group. To further investigate the influence of glucose metabolism and glycemic control on PWV in the normal and hypertensive population, we classified them on the basis of diabetes diagnostic criteria and HbA1c levels. After adjustment for age, gender, BMI, lipid levels, SBP, DBP, WHR, and HR, baPWV in the normotensive group was closely related to different glucose metabolism conditions and glycemic control, whereas glucose status, whether normal or pathological, rarely affected arterial stiffness in hypertensive individuals.

In hypertensive individuals, BP was the main determinant of baPWV despite abnormal glucose status; therefore, the condition of blood vessels may benefit more from intensive antihypertensive treatment than from blood glucose control. This hypothesis is supported by several previous studies. Tomita et al. [23] demonstrated that arterial stiffness was more directly related to BP than to blood glucose levels. The UK Prospective Diabetes Study (UKPDS) followed 1148 hypertensive patients with type 2 diabetes for an average of 8.4 years and observed that tight BP control rather than tight blood sugar control achieved a clinically important reduction in the risk of macrovascular and microvascular complications [24]. Another study by Czernichow et al. [25] demonstrated that BP was the most important metabolic syndrome component in relation to the structure and function of large arteries, thus reflecting our results and emphasizing the importance of BP control in arterial stiffness. Although there are many pharmacological strategies to reduce arterial stiffness, antihypertensive treatment seems to be the most powerful therapy at present [26].

Although our data corroborates those from a majority of previous studies, some controversial results do exist. Bruno et al. [1] recently demonstrated that PWV was significantly higher in hypertensive individuals with diabetes than in hypertensive individuals with normal glucose status. In a study involving 1375 never-treated hypertensive subjects, an association between arterial stiffness and glucose tolerance existed [27]. However, it is noteworthy that these studies did not exclude patients with a history of diabetes or antidiabetic treatment, and this may have affected the results. Moreover, Chen et al. [28] showed that PWV increased with the duration of diabetes in individuals with or without hypertension, suggesting that arterial stiffness gradually worsened with a longer duration of diabetes, which was likely to be another reason for the differences detected in our study that involved patients with no history of diabetes.

The euglycemic hyperinsulinemia clamp studies have proved that 2 h glucose levels are associated with peripheral IR [29], which had an unclear contribution to arterial stiffness. Although many studies concluded that IR was associated with arterial stiffness [30–32], Okada et al. [33] claimed that low insulin levels, which indicate high insulin sensitivity, were also linked to the occurrence of atherosclerosis. Nevertheless, previous studies have not clearly demonstrated whether IR is associated with increased arterial stiffness independent of the clusters of other risk factors. Therefore, we explored the possibility that insulin-sensitivity may influence arterial stiffness in patients with different BP levels. Because HOMA-IR was only based on fasting glucose and insulin levels, we used the Matsuda index as well, which represents whole-body insulin sensitivity in the basal state and after the ingestion of a glucose load. Indeed, our analysis of the normotensive population found a strong association between decreased insulin sensitivity and baPWV, independent of other metabolic factors. However, only the Matsuda index was weakly but significantly associated with baPWV, making it difficult to conclude that IR was an independent risk factor in hypertensive patients.

Moreover, we estimated the association between baPWV and beta-cell function. To our knowledge, the relationship between beta-cell function and arterial stiffness has not been previously explored. Curtis et al. [34] demonstrated that beta-cell function, evaluated by HOMA-*β*, was a significant predictor of incident cardiovascular events, which could be the outcome of arterial stiffness. In our study, HOMA-*β* was significantly correlated with baPWV after adjustment for metabolic factors. Another novel aspect of our study is that not only individuals with diabetes but also those with IR in the absence of diabetes and hypertension are at risk of arterial stiffness. These results suggest that the influence of IR on arterial stiffness is independent of glucose tolerance status. Several clinical studies also support our conclusion to some extent. Insulin sensitizing agents significantly decreased both PWV [35, 36] and IMT [37] of the carotid artery of type 2 diabetes patients, irrespective of their glucose-lowering effects.

The mechanism linking IR and arterial stiffness is not completely understood. Chronic hyperinsulinemia accentuates the activity of the renin-angiotensin-aldosterone axis as well as the expression of angiotensin type 2 receptors in vascular tissue, leading to wall hypertrophy and fibrosis [38, 39]. In addition, IR causes decreased release of nitric oxide and dilation of vasculature, thereby increasing the possibility of damage to the vessel wall [40].

The influence of 30 min or 1 h glucose levels on arterial stiffness has not been clearly defined. In a study from Korea [9], 30 min postchallenge glucose levels, in contrast to FPG and 2 h glucose levels, were a weak but significant determinant of mean baPWV. However, this outcome was not reported in our study. This discrepancy may be due to the different population between studies; the Korean study selected individuals with impaired FPG and acute glucose excursion worse than those seen in the general population.

The role of BMI in hardening of arteries remains controversial, as Tomiyama et al. [41] showed that BMI was positively associated with PWV while Tomiyama et al. [15] and Li et al. [42] reported opposite results. Our findings were consistent with the latter study, where the colinearity between BMI and other cardiovascular risk factors may lead to the negative association. With regard to lipid metabolism, we found that TG and HDL levels were related to baPWV in individuals without hypertension. Only TG levels remained associated with baPWV in the hypertensive group, a finding consistent with that of previous reports [43, 44].

There were limitations to our study. First, because it is a cross-sectional research study, we cannot draw causal conclusions on blood glucose and PWV. Second, OGTT and the insulin index used were not the gold standard to diagnose IR. Third, we evaluated an exclusively Chinese population; therefore, the conclusions may not be applicable to other ethnic groups.

Nonetheless, our study was the first, as per our knowledge, to gauge the impact of glucose metabolism on baPWV in patients with different BP levels. Overall, our data indicated that baPWV was significantly associated with 2 h glucose levels in normotensive individuals, which may be connected with insulin sensitivity. In hypertensive individuals, BP was the dominant factor influencing arterial stiffness; neither glucose level nor islet function had a strong impact on baPWV, suggesting that intensive antihypertensive treatment may be more effective than glycemic control in reducing the incidence of cardiovascular events in these patients. Individuals with abnormal insulin sensitivity are also at increased risk of arterial stiffness, even in the absence of diabetes and hypertension.

## Figures and Tables

**Table 1:** Baseline characteristics of the participants.

	NGT	Pre-DM	DM	*P* value	Age and gender
	(*n* = 1990)	(*n* = 843)	(*n* = 304)		Adjusted *P *
Age (year)	54.71 ± 8.21^a,c^	57.81 ± 8.50	58.18 ± 8.41	<0.001	
Gender (male/female)	708/1082	358/485	147/157	<0.001	
BMI (Kg/m^2^)	23.40 ± 2.89^a,c^	24.78 ± 3.23^b^	25.79 ± 3.03	<0.001	<0.001
WHR	0.86 ± 0.67	0.89 ± 0.63	0.90 ± 630.79	<0.001	<0.001
FPG (mmol/L)	5.35 ± 0.35^a,c^	5.83 ± 0.52^b^	7.45 ± 1.85	<0.001	<0.001
30 min PG	8.52 ± 1.52^a,c^	10.17 ± 1.48^b^	12.82 ± 2.68	<0.001	<0.001
120 min PG	6.04 ± 1.02^a,c^	8.49 ± 1.35^b^	13.89 ± 3.78	<0.001	<0.001
HbA1c (%)	5.60 ± 0.37^a,c^	5.82 ± 0.400^b^	6.85 ± 1.32	<0.001	<0.001
HDL (mmol/L)	1.39 ± 0.35^a,c^	1.32 ± 0.32	1.29 ± 0.29	<0.001	<0.001
LDL (mmol/L)	1.83 ± 0.75^a^	2.93 ± 0.82	3.04 ± 0.82	<0.001	<0.001
TG (mmol/L)	1.38 ± 0.92^a,c^	1.74 ± 1.12^b^	1.97 ± 1.31	<0.001	<0.001
CHOL (mmol/L)	4.85 ± 0.96^a,c^	4.99 ± 1.03	5.11 ± 1.03	<0.001	<0.001
SBP (mmHg)	123.43 ± 15.40^a,c^	130.58 ± 16.13^b^	136.16 ± 18.89	<0.001	<0.001
DBP (mmHg)	76.72 ± 10.13^a,c^	76.69 ± 10.50^b^	82.02 ± 11.70	<0.001	<0.001
baPWV (cm/s)	1424.36 ± 272.94^a,c^	1561.87 ± 330.39^b^	1657.71 ± 327.62	<0.001	<0.001
INS0	11.60 ± 7.26	12.40 ± 8.52	13.73 ± 9.61	<0.001	<0.001
INS30	71.56 ± 54.28^c^	71.59 ± 56.57^b^	44.70 ± 40.01	<0.001	<0.001
INS120	48.74 ± 39.30^a,c^	90.37 ± 69.90	89.60 ± 65.86	<0.001	<0.001

BMI: body mass index, WHR: waist-hip ratio, FPG: fasting plasma glucose, HDL: high-density lipoprotein, LDL: low-density lipoprotein, TG: triglyceride, CHOL: cholesterol, SBP: systolic blood pressure, and DBP: diastolic blood pressure.

INS0: fasting serum insulin, INS30: 30-minute serum insulin, INS120: 120-minute serum insulin, HbA1c: hemoglobin A1c, and baPWV: brachial-ankle pulse wave velocity.

^a^NGT versus Pre-DM, *P* < 0.05.

^b^Pre-DM versus DM, *P* < 0.05.

^c^NGT versus DM, *P* < 0.05.

**Table 2:** Independent relationships between pulse wave velocity and clinical variables in the entire study population and in the individuals with or without hypertension.

	The whole population		Normotension		Hypertension	
	(*n* = 3037)		(*n* = 2475)		(*n* = 652)	
	Beta (95% CI)	*P*	Beta (95% CI)	*P*	Beta (95% CI)	*P*
Age	0.355 (0.323–0.378)	<0.001	0.312 (0.285–0.340)	<0.001	0.523 (0.440–0.606)	<0.001
BMI	−0.086 (−1.116–0.057)	<0.001	−0.060 (−0.090–−0.030)	<0.001	−0.149 (−0.233–−0.065)	0.007
WHR	0.078 (0.047–0.109)	<0.001	0.075 (0.044–0.106)	<0.001		NS
HR	0.171 (0.145–0.197)	<0.001	0.147 (0.121–0.174)	<0.001	0.243 (0.172–0.315)	<0.001
SBP	0.489 (0.449–0.529)	<0.001	0.403 (0.348–0.457)	<0.001	0.474 (0.356–0.539)	<0.001
TG	0.046 (0.011–0.081)	0.001	0.028 (−0.008–0.064)	0.057	0.097 (0.006–0.188)	0.004
HDL		NS	−0.038 (−0.058–−0.018)	0.037		NS
2 h PG	0.052 (0.013–0.090)	0.008	0.046 (0.006–0.087)	<0.001		NS
Adjusted *r* ^2^	0.524		0.402		0.377	

BMI: body mass index, WHR: waist-hip ratio, FPG: fasting plasma glucose, HDL: high-density lipoprotein, LDL: low-density lipoprotein, TG: triglyceride, CHOL: cholesterol, SBP: systolic blood pressure, and DBP: diastolic blood pressure.

INS0: fasting serum insulin, INS30: 30-minute serum insulin, INS120: 120-minute serum insulin.

NS: nonsignificant.

Only significant correlations are listed.

**Table 3:** Independent association of brachial-ankle pulse wave velocity with indices of insulin resistance, insulin sensitivity, and beta-cell function.

	Normotensive			Hypertensive		
	Beta	*P*	Adjusted *r* ^2^	Beta	*P*	Adjusted *r* ^2^
Model 1, age, gender adjusted						

InHOMA-IR	0.508	<0.001	0.261	0.403	0.120	0.198
QUICKI	0.532	<0.001		0.383	0.153	
Matsuda	−0.226	<0.001		−0.086	0.195	
HOMA-*β*	−0.131	<0.001		−0.069	0.141	

Model 2, model 1 + BMI, WHR, HR, and MAP						

InHOMA-IR	0.257	0.043	0.378	0.369	0.125	0.337
QUICKI	0.223	0.023		0.456	0.067	
Matsuda	−0.131	<0.001		−0.143	0.023	
HOMA-*β*	−0.088	<0.001		−0.009	0.839	

Model 3, model 2 + LIPIDS						

InHOMA-IR	0.196	0.076	0.380	0.339	0.160	0.341
QUICKI	0.226	0.046		0.430	0.084	
Matsuda	−0.114	<0.001		−0.126	0.046	
HOMA-*β*	−0.082	<0.001		−0.005	0.903	

HOMA-IR: homeostasis model assessment of insulin resistance, QUICKI: Quantitative Insulin Sensitivity Check Index.

HOMA-IR was log-transformed for comparison.
